# Potential Effects of Repetitive Panfacial Filler Injections on Facelift Surgery and Surgical Outcomes: Survey Results of the Members of The Aesthetic Society

**DOI:** 10.1093/asjof/ojad010

**Published:** 2023-02-06

**Authors:** Iliana Sweis, Lance DeRoss, Shreya Raman, Pravin Patel

**Affiliations:** From the Division of Plastic, Reconstructive, Cosmetic Surgery, University of Illinois at Chicago, Chicago, IL, USA; From the Division of Plastic, Reconstructive, Cosmetic Surgery, University of Illinois at Chicago, Chicago, IL, USA; From the Division of Plastic, Reconstructive, Cosmetic Surgery, University of Illinois at Chicago, Chicago, IL, USA; From the Division of Plastic, Reconstructive, Cosmetic Surgery, University of Illinois at Chicago, Chicago, IL, USA

## Abstract

**Background:**

Facial soft-tissue filler injections are being performed in the United States with increasing popularity.

**Objectives:**

This study aimed to characterize the observations of The Aesthetic Society members regarding the potential impact of repetitive panfacial fillers on the outcomes of subsequent facelift surgery.

**Methods:**

A survey containing closed and open-ended questions was sent to The Aesthetic Society members through email.

**Results:**

The response rate was 3.7%. The majority of the respondents (80.8%) believed that less than 60% of their facelift patients had previous repetitive panfacial filler injections. One half (51.9%) reported that a history of panfacial filler injections increased the difficulty of performing facelifts. A large subset (39.7%) of respondents believed that a history of panfacial fillers increased postoperative complication rates, while the remaining either disagreed (28.9%) or were unsure (31.4%). The most common complications following the facelift surgery included undesirable palpability or visibility of filler (32.7%), compromised flap vascularity (15.4%), and decreased longevity of the lifting effect (9.6%).

**Conclusions:**

This study identified a potential association with repetitive panfacial filler injections and outcomes following facelift surgery, although the exact effect on postoperative outcomes remains unclear. Large prospectively designed studies are needed to capture objective data comparing facelift patients with a history of repetitive panfacial fillers with those facelift patients who have never had injectables. Given the results of The Aesthetic Society members’ survey, the authors encourage careful history-taking to elicit an accurate filler injection record including complications after filler injections, as well as engaging patients in a thorough preoperative discussion regarding the potential of panfacial fillers on the facelift procedure and postoperative outcomes.

**Level of Evidence: 5:**

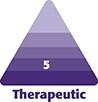

Facial rejuvenation is one of the most commonly requested aesthetic procedures. Historically, facelift surgery has been the standard treatment option for patients presenting with significant soft-tissue laxity and volume loss characteristic of facial aging. With the increase in nonsurgical cosmetic options available, there is a trend toward patients seeking minimally invasive techniques to improve the signs of facial aging. There are numerous nonsurgical modalities that have gained popularity, such as neurotoxins, soft-tissue fillers, lasers, and energy-based soft-tissue tightening devices. Among all nonsurgical procedures, soft-tissue fillers rank second after neurotoxins.^1^ Autologous fat and various synthetic materials are used to enhance facial volume, lift soft tissues, and diminish the appearance of folds and rhytides. The most common synthetic soft-tissue fillers include hyaluronic acid (HA), calcium hydroxylapatite (CaHA), poly-L-lactic acid (PLLA), and polymethylmethacrylate (PMMA). Of these options, HA remains the dominant filler used in the United States.^2^

The increasing popularity of soft-tissue fillers is attributed to the perceived low risk, quick procedural time, minimal recovery period, and immediate results. Despite these benefits, there are still numerous and potentially significant adverse events that can occur. These complications are well documented in the literature and range from minimal, self-limiting outcomes to irreversible injury. Minimal adverse events include ecchymosis, swelling, erythema, granuloma formation, shifting, and contour irregularities. On the extreme end of the spectrum, severe complications include anaphylaxis and vascular injuries such as stroke, blindness, and tissue necrosis.

Even though there has been a trend toward noninvasive facial rejuvenation, surgery remains the standard of care for long-lasting results in the treatment of facial aging. Facelift surgeries have increased by 75% over the past 20 years (2000-2020), even in the presence of exponential growth of nonsurgical options.^3,4^ Therefore, there are more patients undergoing facelift surgery who have a history of repeat injections of panfacial fillers. Despite the well-known complications of soft-tissue fillers, there is a paucity of data about adverse effects of prior repetitive panfacial soft-tissue filler injections on the technical aspects or outcome of facelift surgeries. The aim of this study was to understand the observations of the national aesthetic surgery community regarding facelift surgery in patients who have undergone repetitive panfacial fillers prior to their surgery. The primary outcome was to determine if the aesthetic community believed that a patient's history of repetitive panfacial fillers led to potential difficulties or complications with their facelift procedure, and as a consequence develop more targeted preoperative discussions with these patients and develop safer guidelines when performing surgery on this group.

## METHODS

The authors developed a survey to examine the experiences among members of The Aesthetic Society with patients who have a history of repetitive panfacial fillers and subsequently underwent facelift surgery (Appendix). Our aim was to understand if a history of repetitive panfacial fillers had potential effects on facelift surgery and the postoperative outcomes. This survey contained 10 closed and open-ended questions and was sent through email correspondence to the 4180 members of The Aesthetic Society. The survey was e-mailed twice, first in March 2022 and then in May 2022. Written consent was provided, by which the subjects agreed to the use and analysis of their data. The data were tabulated and examined to analyze the current techniques and perceived outcomes with repetitive panfacial fillers and facelift surgery. Categorial variables were reported as frequencies or percentages.

## RESULTS

**Figure 1.**
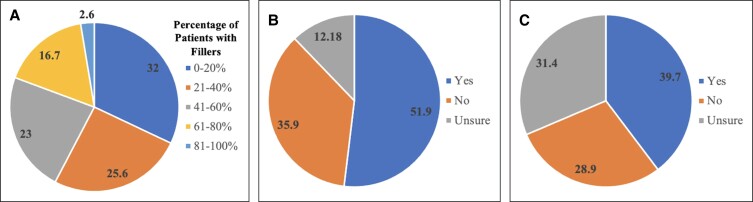
Survey responses from The Aesthetic Society members from the administered questionnaire. (A) Percentage of respondents reporting an estimated frequency of their patients who had undergone previous repetitive panfacial filler injections. Approximately one-third of the respondents believed that 20% or less of their patients had a history of repetitive panfacial fillers, while the remainder of the respondents believed the percentage of filler patients was higher. (B) Percentage of The Aesthetic Society members who reported that a history of previous repetitive panfacial filler injections increased the difficulty of performing a subsequent facelift. Over half of members reported increased difficulty of performing a subsequent facelift procedure. (C) Percentage of The Aesthetic Society members who reported that a history of previous repetitive filler injections increased the rates of complications following a facelift surgery. A larger subset (39.74%) of members believed a prior filler history increased the complication rate.

There were 156 responses, 3.7% of The Aesthetic Society members. Of those that responded, the majority of The Aesthetic Society members (58.33%) performed up to 30 facelift procedures annually, while 20 respondents (12.8%) performed over 60 facelifts per year. Among the members surveyed, the majority preferred a superficial musculoaponeurotic system facelift technique (82.7%), followed by deep plane technique. Nearly all surgeons (98.1%) used HA in their practice, followed by autologous fat (78.8%) as the most common fillers. The majority of the respondents believed that their facelift patients had previously received repetitive panfacial fillers before facelift surgery (Figure 1A).

**Figure 2.**
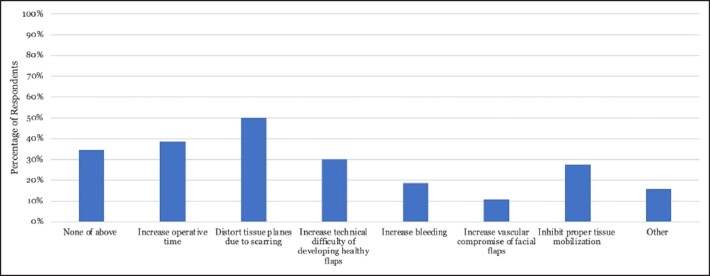
Percentage of The Aesthetic Society members who reported certain effects of prior repetitive panfacial soft-tissue injections on subsequent facelift procedures. Half of respondents reported increased scarring resulting in distorted tissue planes, while approximately 40% of members noted an increased operative time.

However, approximately one half (51.9%) of the responding Aesthetic Society members reported that a history of panfacial fillers increased the difficulty of performing facelifts (Figure 1B). The remaining respondents indicated that it did not make a difference (35.9%) or were unsure (12.2%; Figure 1B). For those who believed there was an increase in difficulty, the most common reason noted was a distortion of the tissue planes due to scarring (50%), followed by technical difficulty of raising healthy flaps (30.1%; Figure 2).

**Figure 3.**
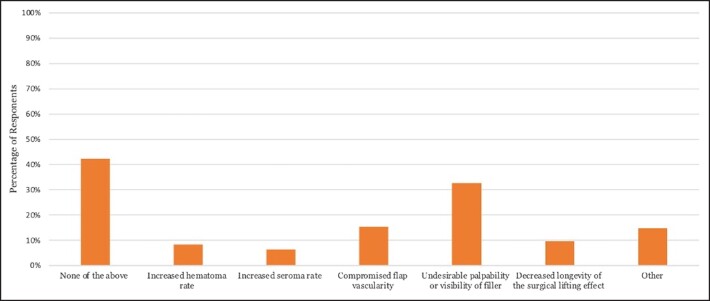
Percentage of The Aesthetic Society members who reported specific complications in facelift patients with repetitive facial filler history. While a larger subset of members (42.3%) reported no specific complications, 32% of members noted increased visibility or undesirable palpability of filler following the procedure.

**Figure 4.**
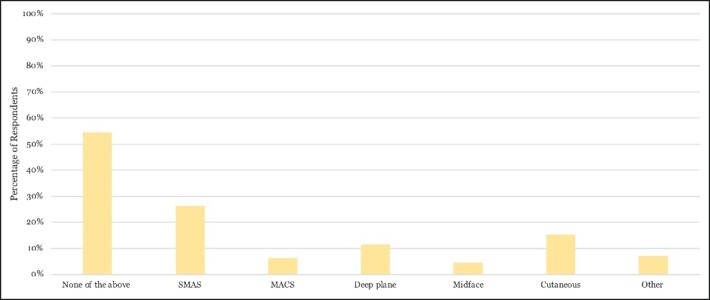
Percentage of The Aesthetic Society members who reported an increased rate of complications with technique of facelift utilized in patients with repetitive facial filler history. Over half of respondents did not believe that the facelift technique affected the rate of complications. MACS, minimal access cranial suspension; SMAS, superficial musculoaponeurotic system.

**Figure 5.**
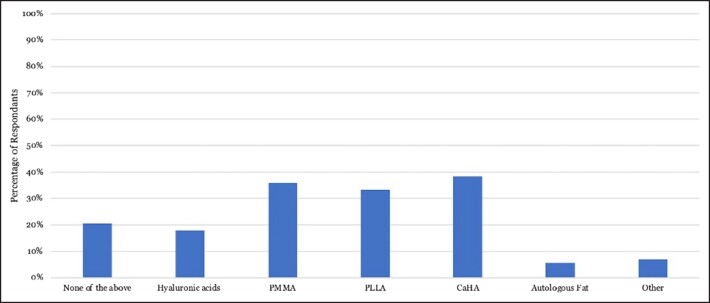
Percentage of The Aesthetic Society members who reported an increased difficulty with subsequent facelift procedure in patients with filler history involving certain filler materials. Polymethylmethacrylate (PMMA), poly-L-lactic acid (PLLA), and calcium hydroxylapatite (CaHA) had similar rates of respondents reporting increased difficulty in a future facelift procedure.

With regards to complications after facelift surgery, 39.7% of The Aesthetic Society members believed that a history of repetitive panfacial fillers increased complication rates compared to 28.9% who disagreed, while 31.4% were unsure (Figure 1C). The most common complications reported included the undesirable palpability or visibility of filler (32.7%), compromised flap vascularity (15.4%), and decreased longevity of the lifting effect (9.6%; Figure 3). Over half of respondents (55%) did not believe that the facelift technique utilized in patients with prior filler history affected the rate of complications during the procedure (Figure 4). Rather, of the various types of filler product used, 38.5% believed that CaHA and 35.9% believed that PMMA caused the most problems with subsequent facelift surgery (Figure 5). Approximately 5% of The Aesthetic Society respondents found that facial autologous fat injections increased the difficulty of a future facelift procedure (Figure 5).

## DISCUSSION

The Aesthetic Society published its annual survey results, which revealed an overall 55% increase in all facial aesthetic procedures from 2020 to 2021. For facelift surgery and dermal fillers, there was a 54% and 42% increase, respectively.^5^ The rise of video-based communication in the era of the COVID-19 pandemic has fueled this increased demand for cosmetic facial procedures.

As nonsurgical facial rejuvenation has been increasing in popularity, the likelihood of patients with a history of repetitive panfacial fillers prior to facelift surgery will continue to rise. Jacono et al found that 32% (26/82) of their patients surveyed underwent injectable treatments at some point prior to facelift with a mean of 7 rounds of treatment prior to surgery.^5^ Of note, their data was collected from 2003 to 2013; therefore, in this current landscape of increasing facial rejuvenation procedures, there is likely a higher percentage of patients with a history of repetitive panfacial fillers seeking facelift surgery.^3,4^ This trend is expected to continue with increased availability of the ever-evolving filler products.

The perceptions of our survey respondents regarding this topic varied greatly. Approximately one-third of the respondents believed that 20% or less of their patients had a history of repetitive panfacial fillers, while very few respondents (2.6%) believed that most of their patients (81%-100%) received panfacial fillers at some point prior to surgery. This variation in perception is likely due to differences in practice patterns among surgeons and partly due to the difficulty in obtaining an accurate patient history due to patients' recall bias. In some surgical practices, the patient coordinator may be responsible for obtaining the patient history, and in some cases, less emphasis may be placed on obtaining a filler history. Also, it is not uncommon for a patient to be uncertain of which filler product (HA vs CaHA vs PLLA) has been injected in the past or recall which areas have been previously treated, especially if they have undergone multiple injections by different injectors over time.

The most reported (32.7%) complication listed on the survey was undesirable palpability or visibility of filler. The use of soft-tissue fillers is known to potentially lead to a wide spectrum of complications. Minimal and self-limited complications are more common and include ecchymosis, edema, and erythema. More significant events that may or may not be self-limited include overcorrection, contour irregularities, visibility, and granuloma formation. Lastly, the most severe complications include tissue necrosis, visual impairment, and anaphylaxis.^6^ It is difficult to determine the overall incidence of complications following injection of soft-tissue fillers given their widespread use by multiple types of practitioners and their off-label usage. Multiple published studies have analyzed information from company-based data systems, national societies and drug adverse event reporting systems and concluded an estimated incidence of filler complications of 0.0001% to 0.15%.^6–9^ However, these statistics are only from reported complications. Many complications go unreported as they are not being performed by properly qualified injectors who recognize the need for transparency. Therefore, given the reported low incidence of facelift complications and even lower reported incidence of filler complications, it may be challenging to determine the actual degree of impact and if there is a cause and effect relationship.

Even though it may be difficult to determine if repetitive panfacial fillers have an effect on facelift outcomes, at least half (51.9%) of the survey respondents believed that they increased the difficulty of performing the facelift, while approximately 40% felt that a history of panfacial fillers increased the potential for complications after facelift surgery. Dermal fillers are known to occasionally cause an immunological reaction leading to inflammatory nodules, granulomas, cutaneous vasculitis, and induration.^10–12^ In most cases, these complications are self-limiting, but can be more persistent and difficult to treat with semipermanent or permanent fillers, such as CaHa, PLLA, and PMMA.^6^ Previous studies have demonstrated that the incidence of delayed nodules/granulomas is between 0.02% and 15%.^8,12–14^ If these complications persist, intralesional steroids, or incision and drainage may be indicated.^15–17^ If left untreated, it is possible that these nodules can lead to a more difficult dissection during surgery or unfavorable outcomes. Furthermore, there is a possibility that in some patients with ptotic skin or thicker subcutaneous tissue, nodules or granulomas may not be evident until the facelift surgery. In these scenarios, once the redundant skin is removed and re-draped, the uneven contours of deeper, untreated nodules may become more evident.

**Figure 6.**
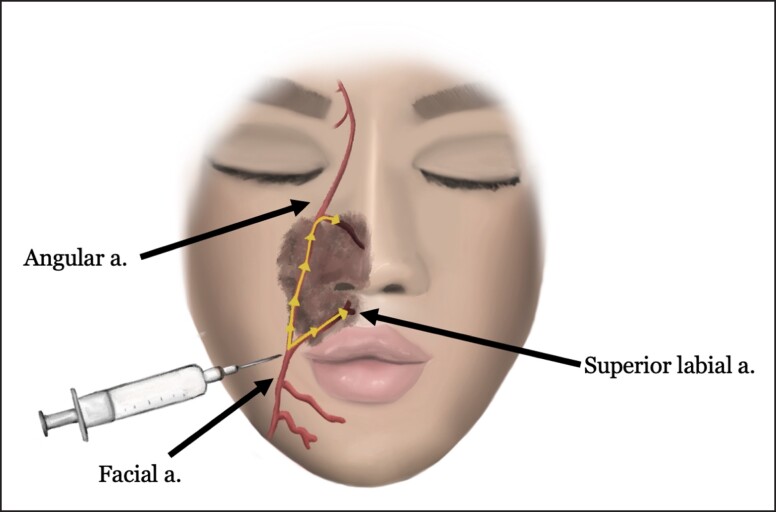
Injection of filler in the nasolabial fold or peri-oral region has potential of vascular injury to more superficial branches of the facial artery resulting in dermal ischemia along the nasolabial fold, upper lip, nasal ala, and nasal tip.

**Figure 7.**
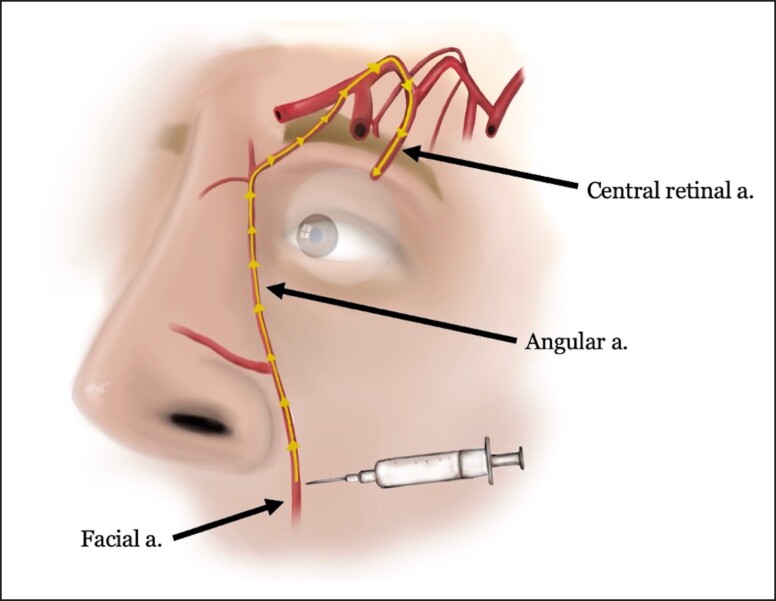
Injection of filler along the upper nasolabial fold has potential for vascular injury to the angular artery with migration of product (arrows) into the ophthalmic artery and central retinal artery, potentially causing blindness.

The second most common complication cited from our survey was compromised flap vascularity (15.4%). The vascular supply of the face is extremely robust with an elaborate network of connections between multiple feeding vessels. A detailed understanding of the location and depth of each vessel is crucial to avoid vascular complications during both facelift surgery and filler injections. Vascular complications after filler injections can occur through multiple routes: injection of filler material intravascularly leading to an embolic event, direct injury of a vessel from the needle, or filler deposited surrounding a vessel leading to external compression.^18,19^ If vascular injury has occurred, it may lead to dermal ischemia and present as livedo reticularis, erythromelalgia, ulceration, or frank dermal infarct.^20^ More common areas of vascular injury include filler placement in the nasolabial fold or peri-oral regions. Accidental placement of filler in branches of the facial artery located superficially in these regions may result in dermal ischemia along the nasolabial fold, upper lip, nasal ala, and nasal tip (Figure 6). As a particularly devastating consequence, flow of product injected into the superficial branches of the facial artery could cause blindness with retrograde migration of a bolus to the facial artery, coursing the angular artery and ending in the central retinal artery^21^ (Figure 7). Apart from this potentially permanent outcome, due to the extensive blood supply to the facial skin, most dermal vascular ischemic injuries usually heal without significant long-term sequelae.^21^ Furthermore, many patients who suffered these vascular injuries do not recall the true nature of the injury and frequently neglect to mention it during a face lift consultation. The lack of physical evidence of the dermal injury and poor recollection on the part of the patient make it impossible for the future plastic surgeon to identify the past dermal vascular changes that may compromise the vascularity of the facial flap.

Due to possibility for complications following a facelift surgery with a history of fillers, there are critical steps in a surgeon's preoperative evaluation of a potential facelift candidate. It is not only important to include a thorough investigation of a patient's history of filler treatments, but it is also imperative to clarify whether the patient experienced skin discoloration after filler treatment, which could be indicative of a history of vascular injury. Furthermore, the location of injury should also be ascertained, as the vascularity of the skin flaps raised during a facelift could be affected, and potentially increase the risk of wound healing complications postoperatively from a facelift. It may be difficult to obtain this history from a patient as most patients have undergone filler placement by different injectors, had different products injected, and therefore, often cannot recall what was used and where it was placed in their face. It may be prudent to discuss the potential increased risks with these patients at their preoperative visit when obtaining consent.

Another consideration of the presurgical evaluation is the appropriate timing of facelift surgery following uncomplicated panfacial filler injections. At this time, HA remains the most commonly used soft-tissue filler, followed by CaHA, PLLA, and fat grafting.^3^ Each material has a distinct composition and duration of effect. Depending upon the density used, HA is expected to remain in the tissue from 4 to 12 months.^6^ However, not infrequently, HA has been found in certain regions of the face for longer periods than this reported lifespan. Hyaluronidase may be repetitively used to dissolve much of the HA, though residual HA may still be present due to product migration or existence in multiple facial tissue planes. The CaHa and PLLA are considered semipermanent and may remain in the tissue from 1 to 2 years.^6^ Lastly, PMMA is considered permanent. Therefore, an understanding of the properties of each filler material plays an important role during preoperative discussions with potential facelift patients. If there is any uncertainty in a patient's filler history or concern of previous filler complications, then it may be advisable for the patient to wait longer than the expected duration of effect before undergoing a facelift. Patients should also be informed of the possibility that filler may still be present beyond its predicted lifespan, which may alter the long-term results of a facelift procedure. Computed tomography, ultrasound, or magnetic resonance imaging, while possibly helpful in identifying presence of filler in select cases, are not the standard of care in preoperative planning for a facelift procedure. In the least, it would be important to discuss the possibility of suboptimal results given a filler history with the patient and specifically include these risks in an informed consent.

There are several inherent limitations to the survey. Given the lower response rate from the survey, it is possible that the results may not represent the predominant surgical practices and experiences of facial aesthetic surgeons. It is also possible that the survey was sent to a large number of The Aesthetic Society members who do not perform a significant number of facelifts, which may have led to a dilutional effect and the low response rate. Since the survey was sent only to The Aesthetic Society members, there are surgeons outside the society who do not have their perspectives reflected in these results. Additionally, questions related to the respondents' opinions regarding potential facelift complications related to fillers or speculation on their patients' history of filler injections are subjective and therefore, open to bias.

To objectify and further analyze some of our findings, we would conduct a prospective study and invite experienced plastic surgeons who perform 30 or more face lifts per year to participate. Face lift patients would be divided into 2 groups: filler-naïve patients and panfacial filler patients who have had 2 or more rounds of facial fillers performed within the 2 years prior to their facelift. The surgeons would then assess each surgery by noting exact length of the procedure; degree of bleeding; and ease of raising the facial flap. The immediate postoperative course would be assessed based on presence of compromised skin flaps, hematomas, or bunching of the facial tissues. The long-term postoperative course would be assessed by following the patients at regular intervals for a period of 2 years and noting the length of time for evidence of recurrent tissue relaxation as assessed by the surgeon and the patient. This prospective study design may help elucidate a more objective analysis of what our survey results have revealed.

## CONCLUSIONS

The outcome of this survey identified that there may be a potential association between repetitive panfacial filler injections and outcomes following facelift surgery, although the exact effect on postoperative complications remains unclear. Future large, prospectively designed studies are needed to capture objective data comparing facelift patients with history of repetitive panfacial fillers with those who have never had injectables. These data have the potential to impact surgical practice and presurgical planning for facial rejuvenation. At the very least, it will help surgeons provide a more detailed informed consent when patients are undergoing facelift surgery after undergoing repetitive panfacial filler injections. Overall, it appears that the outcomes following facelift surgery in patients with a history of repetitive panfacial fillers warrant additional consideration in the literature, as the majority of respondents in this survey identified an increased technical difficulty of the surgery with at least one-third reporting an increase in postoperative complications.

## Supplementary Material

ojad010_Supplementary_Data
