# Correction to: Potential Effects of Repetitive Panfacial Filler Injections on Facelift Surgery and Surgical Outcomes: Survey Results of the Members of The Aesthetic Society

**DOI:** 10.1093/asjof/ojad025

**Published:** 2023-03-27

This is a correction to: Iliana Sweis, MD, Lance DeRoss, MD, Shreya Raman, MD, Pravin Patel, MD, Potential Effects of Repetitive Panfacial Filler Injections on Facelift Surgery and Surgical Outcomes: Survey Results of the Members of The Aesthetic Society, *Aesthetic Surgery Journal Open Forum*, Volume 5, 2023, ojad010, https://doi.org/10.1093/asjof/ojad010

In the originally published version of this manuscript, there was an typographical error in first author's first name. This should read: “Iliana Sweis“ instead of: “Liana Sweis“.

The publisher apologizes for this error, which has been been corrected within the article.

