# Supplementary figures and images for: Potential Effects of Repetitive Panfacial Filler Injections on Facelift Surgery and Surgical Outcomes: Survey Results of the Members of The Aesthetic Society
Source: Aesthet Surg J Open Forum. 2023 Feb 6;5:ojad010. doi: 10.1093/asjof/ojad010 (PMC9969530; doi:10.1093/asjof/ojad010)

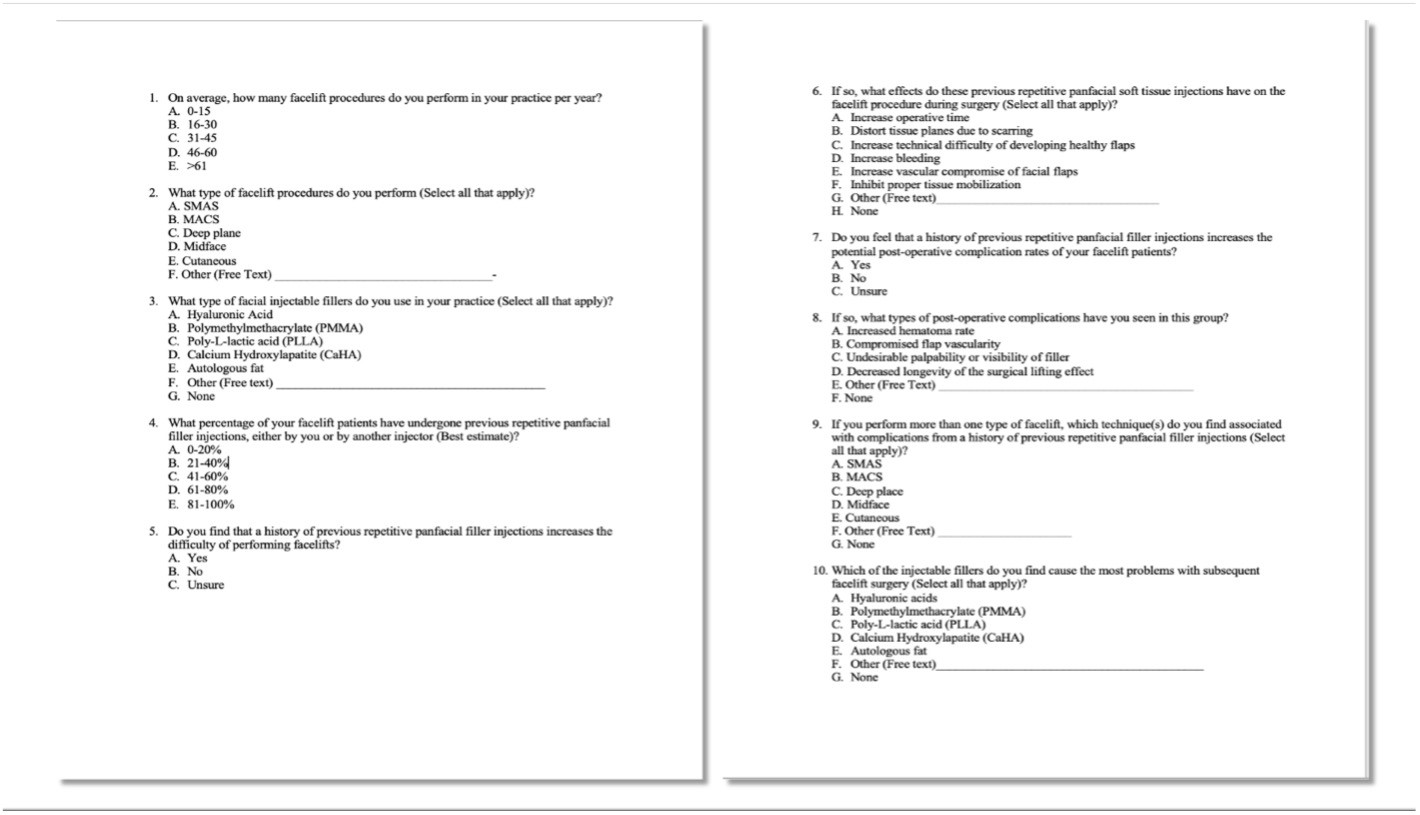

Supplement: ojad010_Supplementary_Data [file ojad010_supplementary_data.jpeg]
